# Genomic population structure and inbreeding history of Lake Superior caribou

**DOI:** 10.1002/ece3.10278

**Published:** 2023-07-07

**Authors:** Kirsten Solmundson, Jeff Bowman, Micheline Manseau, Rebecca S. Taylor, Sonesinh Keobouasone, Paul J. Wilson

**Affiliations:** ^1^ Environmental & Life Sciences Graduate Program Trent University Peterborough Ontario Canada; ^2^ Wildlife Research and Monitoring Section, Ontario Ministry of Natural Resources and Forestry Trent University Peterborough Ontario Canada; ^3^ Landscape Science and Technology Division Environment and Climate Change Canada Ottawa Ontario Canada; ^4^ Biology Department Trent University Peterborough Ontario Canada

**Keywords:** caribou, conservation genomics, inbreeding, population structure, whole genomes

## Abstract

Caribou (*Rangifer tarandus*) have experienced dramatic declines in both range and population size across Canada over the past century. Boreal caribou (*R. t. caribou*), 1 of the 12 Designatable Units, has lost approximately half of its historic range in the last 150 years, particularly along the southern edge of its distribution. Despite this overall northward contraction, some populations have persisted at the trailing range edge, over 150 km south of the continuous boreal caribou range in Ontario, along the coast and nearshore islands of Lake Superior. The population history of caribou along Lake Superior remains unclear. It appears that these caribou likely represent a remnant distribution at the trailing edge of the receding population of boreal caribou, but they may also exhibit local adaptation to the coastal environment. A better understanding of the population structure and history of caribou along Lake Superior is important for their conservation and management. Here, we use high‐coverage whole genomes (*N* = 20) from boreal, eastern migratory, and barren‐ground caribou sampled in Manitoba, Ontario, and Quebec to investigate population structure and inbreeding histories. We discovered that caribou from the Lake Superior range form a distinct group but also found some evidence of gene flow with the continuous boreal caribou range. Notably, caribou along Lake Superior demonstrated relatively high levels of inbreeding (measured as runs of homozygosity; ROH) and genetic drift, which may contribute to the differentiation observed between ranges. Despite inbreeding, caribou along Lake Superior retained high heterozygosity, particularly in genomic regions without ROH. These results suggest that they present distinct genomic characteristics but also some level of gene flow with the continuous range. Our study provides key insights into the genomics of the southernmost range of caribou in Ontario, beginning to unravel the evolutionary history of these small, isolated caribou populations.

## Graphical abstract

We used high‐coverage whole genomes (*N* = 20) from several caribou ecotypes to investigate population structure and inbreeding histories. Caribou from the Lake Superior range demonstrated high levels of genetic drift and inbreeding and were differentiated from the northern continuous range, but we also found evidence of gene flow between ranges. This study provides key insights into the genomics of the southernmost range of caribou in Ontario, beginning to unravel the evolutionary history of these small, isolated populations.

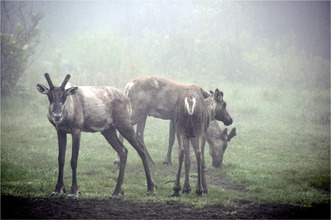

## 1 INTRODUCTION

The caribou (*Rangifer tarandus*), an iconic Canadian species, has experienced dramatic declines in both range and population size over the past century, raising conservation concerns (Festa‐Bianchet et al., 2011; Laliberte & Ripple, 2004). Caribou are ecologically diverse and central to the culture and livelihood of Indigenous peoples (Festa‐Bianchet et al., 2011; Polfus et al., 2016). Caribou diversity is described by several subspecies and ecotypes, which differ in morphology and behavior; for example, barren‐ground caribou (*R. t. groenlandicus*) congregate in large, migratory groups on the tundra (COSEWIC, 2016). Conversely, the woodland subspecies (*R. t. caribou*) has several ecotypes associated with different habitats, such as caribou found in the mountains across western Canada (COSEWIC, 2014b), the eastern migratory caribou that migrate between the boreal forest and the tundra in eastern Canada (COSEWIC, 2017b), and boreal caribou that are more sedentary and found throughout the boreal forest (COSEWIC, 2014a). The diversity found in caribou has resulted in the recognition of 12 Designatable Units (DUs) by the Committee on the Status of Endangered Wildlife in Canada (COSEWIC, 2011). Despite this diversity, all extant caribou in Canada have been recommended for listing as Species‐at‐Risk (Endangered, Threatened, or Special Concern) by COSEWIC (2014a, 2014b, 2015, 2016, 2017a, 2017b). The species is classified as Vulnerable by the IUCN throughout its circumpolar range (Gunn, 2016).

The declining trends observed in caribou populations across Canada have raised conservation concerns, as small and isolated populations are more prone to inbreeding and may eventually fall into an “extinction vortex” and become extirpated (Festa‐Bianchet et al., 2011; Gagnon et al., 2019; Gilpin & Soule, 1986). The extent of inbreeding likely varies among populations; however, especially in the context of historical population fluctuations and recent declines. Additionally, recent phylogenomic analyses showed that the evolutionary lineages of caribou are not concordant with current DUs (Taylor et al., 2022), presenting further insights for conservation and management.

**FIGURE 1:**
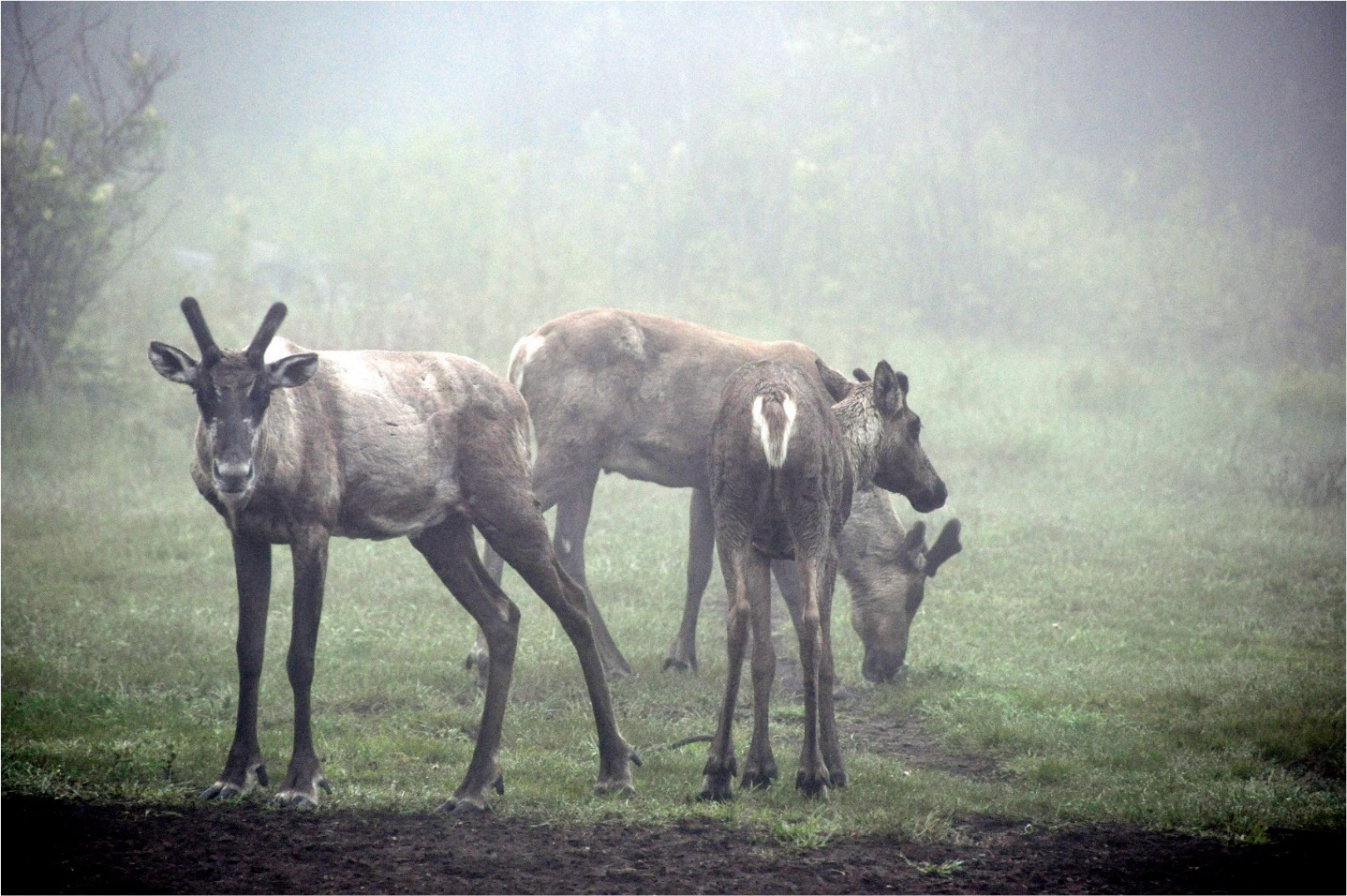
Caribou (*Rangifer tarandus*) surrounded by early morning mist on Michipicoten Island, Lake Superior, Ontario, Canada. Photo by Andy Silver (Ontario Ministry of Natural Resources and Forestry).

**FIGURE 2:**
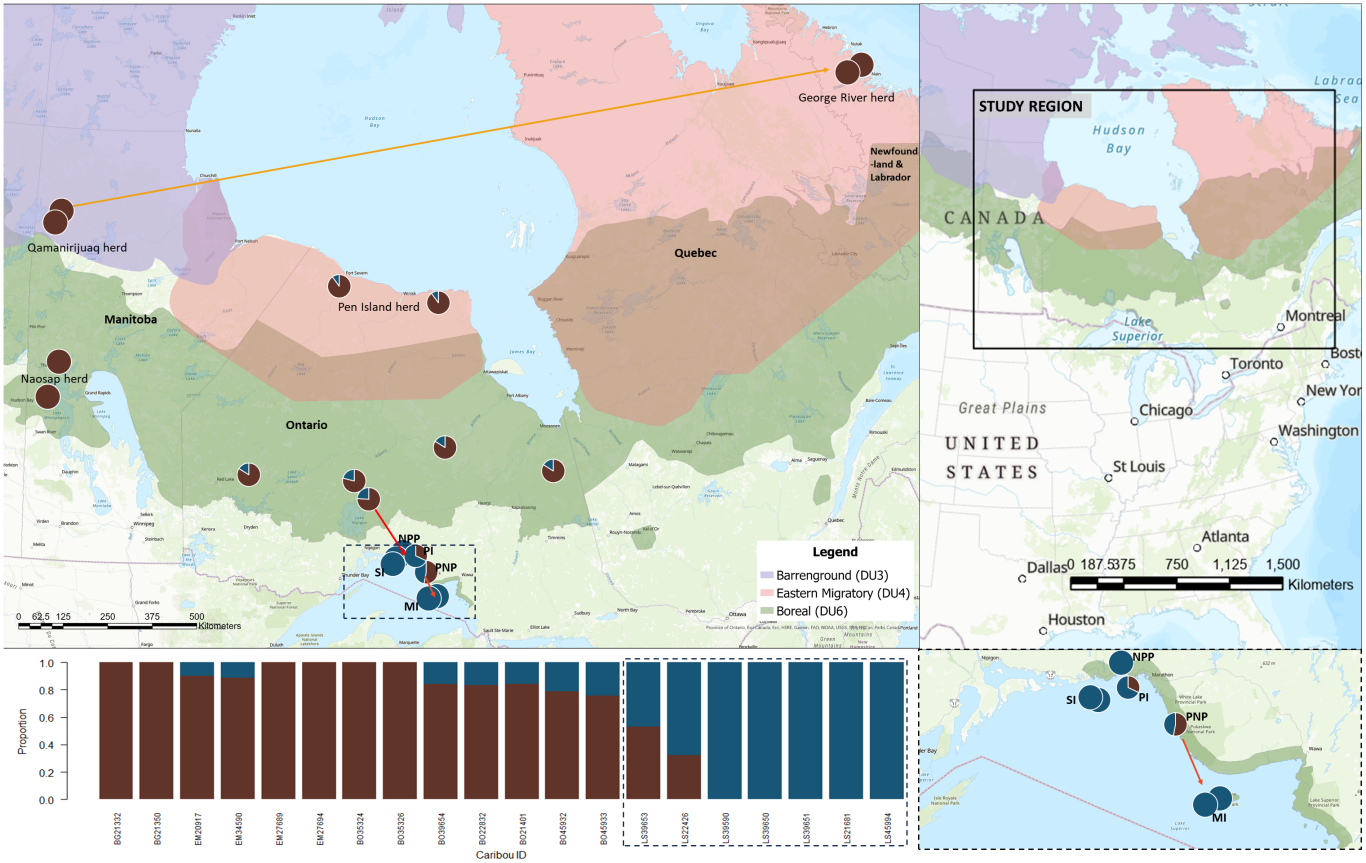
Sample sites of caribou (*N* = 20) in this study. Background colors show the ranges of three of the Canadian Designatable Units (DUs) included: barren‐ground, eastern migratory, and boreal. Circles on map indicate sample locations and the colors indicate individual population assignment proportions under the best supported model of *K* = 2. The arrows on the map indicate migrations modeled by Treemix. The Lake Superior region is indicated by dashed borders; abbreviated labels indicate site names: Pukaskwa National Park (PNP), Neys Provincial Park (NPP), Pic Island (PI), The Slate Islands (SI), and Michipicoten Island (MI). *X*‐axis label indicates individual IDs.

Declines in caribou ranges and population sizes have resulted in small and isolated populations, particularly within the southern mountain and boreal ecotypes (COSEWIC, 2014a, 2014b). A recent microsatellite study revealed genetic erosion, a decrease in connectivity, and an increase in inbreeding along the southern continuous range edge of boreal caribou in Ontario and Manitoba (Thompson et al., 2019). In Ontario, the southern continuous range edge of boreal caribou has been contracting northward for over a century, primarily due to anthropogenic habitat disturbance (Schaefer, 2003). Boreal caribou rely on dense forest for sufficient forage and to avoid wolf predation when calving, and thus are limited by habitat loss and fragmentation in parts of their historic range (Festa‐Bianchet et al., 2011). This range loss has resulted in isolated populations on the trailing range edge that have managed to persist along the coast and on nearshore islands of Lake Superior (Figure 1), over 150 km south of the continuous range edge (Figure 2; Ontario Ministry of Natural Resources, 2009; Schaefer, 2003). The recent history (1900s‐present) of caribou along Lake Superior is well documented (e.g., Bergerud, 1985, 2001; Bergerud et al., 2007, 2014; Carr et al., 2012; Ontario Ministry of Natural Resources and Forestry, 2018; Patterson et al., 2014); however, their deeper evolutionary history remains unclear. A recent microsatellite study suggested some genetic structure within the region and detected low levels of gene flow between Lake Superior caribou from Pukaskwa National Park and caribou farther north in the continuous range (Drake et al., 2018).

Conservation management typically assumes populations along the range periphery are less likely to persist than those in the range core; however, an extensive multispecies review revealed that most species persisted in the periphery of their historical geographical ranges (Channell & Lomolino, 2000). Notably, when a species' historical range included both mainland and island sites, population persistence was highest on islands, even when island habitat patches were smaller than those on the mainland (Channell & Lomolino, 2000). In general, islands harbor greater proportions of threatened species than expected when compared to mainland habitats (Ricketts et al., 2005; Spatz et al., 2017). However, this disparity is often because islands represent the last sites to be disturbed by anthropogenic factors, allowing remnant populations to persist even when populations on the mainland have been extirpated (Lomolino & Channell, 1998). Another review demonstrated that as ranges contract, small and isolated populations along the rear edge can become disproportionately important for the species' survival and evolution (Hampe & Petit, 2005). Small population size and prolonged isolation reduces within population genetic diversity; however, trailing edge populations also demonstrate disproportionately high levels of genetic differentiation when compared to nearby populations (Hampe & Petit, 2005). The conditions created by trailing edge dynamics can encourage selection for local adaptation and reduced gene flow, ultimately resulting in remarkably distinct populations (Castric & Bernatchez, 2003; Hampe & Petit, 2005; Pérez‐Tris et al., 2004). Both drift and local adaptation can contribute to the unique characteristics of rear edge populations, and these effects can be difficult to disentangle (Prentice et al., 2017). Regardless, rear edge populations face a high risk of local extinction, especially when regional population dynamics such as immigration are impeded by isolation (Hampe & Petit, 2005).

In this study, we used high‐coverage whole genome sequences from 20 caribou to investigate population structure and inbreeding in small and isolated populations of boreal caribou from the Lake Superior range, boreal caribou from the continuous caribou range of Ontario and Manitoba, eastern migratory caribou from Ontario and Quebec, and barren‐ground caribou from northern Manitoba (Figure 2). We expected that population clustering among caribou would broadly reflect the ecotypes and sample locations. However, previous research suggested that eastern migratory caribou originated from introgression between barren‐ground and boreal caribou (Klütsch et al., 2016), and a subsequent study indicated introgression has occurred among the barren‐ground, eastern migratory, and boreal ecotypes (Taylor et al., 2020). Thus, this historic exchange of genetic material may be detected as migration or gene flow.

Caribou in the Lake Superior range persist in small and apparently isolated island populations (Drake et al., 2018; Schaefer, 2003), and we tested the hypothesis that these caribou exhibit characteristics of a trailing edge, including effects of genetic drift, low within‐population diversity, and high differentiation from the continuous range. Therefore, we expected to observe high levels of inbreeding in the Lake Superior range and a high degree of differentiation with caribou from the continuous range. We expected to detect lower levels of inbreeding in boreal caribou from the continuous range of Ontario and Manitoba, as well as in the eastern migratory caribou; herds that have experienced recent declines but are not as small and isolated as the Lake Superior range (COSEWIC, 2014a, 2017b). Further, we predicted barren‐ground caribou from the Qamanirijuaq population ranging over northern Manitoba and Nunavut (Figure 2) would have the lowest inbreeding estimates, as they occur in large populations that have not experienced dramatic historical or recent declines (COSEWIC, 2016). The lengths of genomic regions produced by inbreeding, called runs of homozygosity (ROH), indicate how recently inbreeding occurred, as continuous stretches of ROH are broken up during successive mating events (Ceballos et al., 2018). Thus, we predicted we would find the longest ROH in caribou from the Lake Superior range, reflecting recent inbreeding caused by anthropogenic range contraction (Schaefer, 2003), but we may also find short ROH, representing historical inbreeding events, where long ROH have been broken up through mutation and recombination (Ceballos et al., 2018).

## 2 METHODS

### 2.1 Caribou sampling

We sampled caribou from herds that differed in evolutionary history, demographic history, and extent of isolation. Broadly, caribou in North America can be divided into two lineages: the North American Lineage (NAL), which encompasses boreal and eastern migratory caribou (*R. t. caribou*), and the Berigan‐Eurasian Lineage (BEL), represented in this study by barren‐ground caribou (*R. t. groenlandicus*; Klutsch et al., 2012; Polfus et al., 2017; Taylor et al., 2020). Boreal caribou samples (muscle, hide, hair, fecal pellet, and shed antler; Table S1) were collected from the southern caribou range of Ontario by provincial biologists and sequenced for the study and can be retrieved from the National Center for Biotechnology Information (NCBI) under the BioProject accession no. PRJNA 984705. We also included previously sequenced whole genome raw reads (Taylor et al., 2020; BioProject accession no. PRJNA 634908).

We included seven samples from the Lake Superior range in Ontario (Table 1): two samples from Michipicoten Island (LS39650, LS39651), two from the Slate Islands (LS21681, LS45994), one from the mainland area near Neys Provincial Park (LS39590), one from Pic Island of Neys Provincial Park (LS22426), and one from Pukaskwa National Park (LS39653). Over the past four decades, caribou herds along the coast and islands of Lake Superior have steadily declined and become increasingly isolated from the continuous caribou range of Ontario (Ontario Ministry of Natural Resources and Forestry, 2018; Patterson et al., 2014; Shuter et al., 2016). There have been no caribou observed in the coastal Pukaskwa National Park in recent years, although some caribou have managed to persist on small islands. The island populations were founded by very few individuals, but in the absence of predation, they increased to high densities prior to recent declines. For instance, Michipicoten Island was founded by a single resident male plus eight caribou that were relocated from the Slate Islands in 1982–1989, and subsequently grew to an estimated population of 680 caribou by 2010 (Ontario Ministry of Natural Resources and Forestry, 2018). However, the population quickly collapsed when predation pressure was introduced by wolves who immigrated to the island via an ice bridge (in 2014), prompting a relocation of some of the few remaining caribou to the Slate Islands in early 2018 (Ontario Ministry of Natural Resources and Forestry, 2018). The Slate Islands once had the highest density caribou population in North America (Bergerud et al., 2007); however, over the past decade the population had also collapsed and was functionally extirpated at the time of relocation (i.e., there appeared to be only two resident bulls remaining). Our study includes two samples from Michipicoten Island, collected shortly after the population began to decline due to new predation pressure (2015, 2016). From the Slate Islands, we included one sample collected prior to the recent population declines (2009), and another collected shortly before caribou were relocated from Michipicoten Island (2017).

**TABLE 1:** Information for each caribou included in this study: sample individual reference number, subspecies classification, Canadian Designatable Unit, and approximate sampling location indicating herd or region sampled and the province.

Sample ID	Subspecies	Designatable unit	Sample region	Year	Mean depth	*F*_ROH (PLINK)_	Mean length ROH _(PLINK)_	*F*_ROH (ROHan)_	Mean length ROH _(ROHan)_
BG21332	*R. t. groenlandicus*	Barren‐ground	Brochet Junction area, MB	2008	38×	0.003	381	0.001	625
BG21350	*R. t. groenlandicus*	Barren‐ground	Brochet Junction area, MB	2007	38×	0.002	372	0.002	1060
EM20917	*R. t. caribou*	Eastern migratory	Fort Severn, ON	NA	36×	0.023	376	0.009	688
EM34590	*R. t. caribou*	Eastern migratory	Pen Islands, ON	1992	37×	0.037	488	0.025	1320
EM27689	*R. t. caribou*	Eastern migratory	George River, NL	2008	38×	0.048	509	0.021	1930
EM27694	*R. t. caribou*	Eastern migratory	George River, NL	2008	39×	0.022	356	0.002	833
BO35324	*R. t. caribou*	Boreal	The Pas, MB	2008	35×	0.071	582	0.058	1170
BO35326	*R. t. caribou*	Boreal	Snow Lake, MB	2009	38×	0.041	561	0.013	1260
BO39654	*R. t. caribou*	Boreal	Cochrane, ON	2009	38×	0.048	531	0.014	1120
BO22832	*R. t. caribou*	Boreal	Hearst, ON	2009	19×	0.030	410	0.021	947
BO21401	*R. t. caribou*	Boreal	Red Lake, ON	2008	10×	0.028	389	0.017	799
BO45932	*R. t. caribou*	Boreal	Nipigon, ON	2011	13×	0.030	448	0.022	1010
BO45933	*R. t. caribou*	Boreal	Nipigon, ON	2012	18×	0.053	556	0.043	1470
LS39653	*R. t. caribou*	Boreal	Pukaskwa National Park, ON	1999	40×	0.420	1005	0.171	2320
LS22426	*R. t. caribou*	Boreal	Pic Island, ON	<2008	10×	0.052	522	0.040	1370
LS39590	*R. t. caribou*	Boreal	Neys area, ON	2011	35×	0.250	884	0.225	3160
LS39650	*R. t. caribou*	Boreal	Michipicoten Island, ON	2015	37×	0.215	800	0.161	2330
LS39651	*R. t. caribou*	Boreal	Michipicoten Island, ON	2016	40×	0.212	861	0.081	1790
LS21681	*R. t. caribou*	Boreal	Slate Islands, ON	2009	10×	0.188	711	0.179	3250
LS45994	*R. t. caribou*	Boreal	Slate Islands, ON	2017	17×	0.251	865	0.248	3810

*Note*: Mean depth refers to the average depth of coverage from filtered whole genome BAM files. Inbreeding was quantified as the proportion of the genome in Runs of Homozygosity (*F*_ROH_) identified with PLINK and ROHan. Table values reflect ROH measured in kb, identified under the 250 kb size class and more relaxed set of parameters tested for each method.

Abbreviations for Canadian provinces: MB, Manitoba; NL, Newfoundland and Labrador; ON, Ontario.

We also selected seven samples from the continuous boreal caribou range in Ontario (BO21401, BO22832, BO39654, BO45932, and BO45933) and Manitoba (BO35324 and BO35326). Within the eastern migratory ecotype, we included two samples from the George River herd (EM27689 and EM27694) and two from the Pen Islands herd (EM20917 and EM34590). The George River herd has experienced a dramatic population decline over recent decades from approximately 823,000 individuals in 1993 (Couturier et al., 1996), to approximately 8900 individuals in 2016 (Gagnon et al., 2019); the samples included in this study were obtained in 2008 after the population had already begun to decline. The Pen Islands herd in northern Ontario was estimated to contain 16,638 individuals in 2011 (COSEWIC, 2017b). Notably, the George River and Pen Islands herds are geographically isolated from each other (Figure 2) and recent research has revealed a divergent evolutionary history between these two populations (Taylor et al., 2020). We also included two barren‐ground caribou samples from the Qamanirijuaq herd (BG21332, BG21350), a large population (estimated to contain 264,661 individuals in 2014) that has not experienced dramatic historical or recent declines (COSEWIC, 2016).

### 2.2 Genome sequencing, assembly, and quality control

DNA was extracted using the Qiagen DNeasy Kit, following the manufacturer's protocols (Qiagen). The extracted DNA was quantified using a Qubit system (Thermo Fisher Scientific) to ensure all samples were above the minimum threshold required for next‐generation sequencing (20 ng/μL). The extracted DNA was then sent to The Centre for Applied Genomics (TCAG), at The Hospital for Sick Children (Toronto, ON). An Illumina library prep kit (Illumina) with an insert size of 350 bp was used to fragment the DNA and apply sequencing adapters. Samples were sequenced on the Illumina HiSeq X platform, yielding paired‐end 150 bp sequence reads. The raw sequence reads are available through the National Center for Biotechnology Information (NCBI) BioProject accession numbers PRJNA 634908 and PRJNA 984705 (Table S1).

We conducted all bioinformatic analyses using cloud computing resources from Compute Canada (RRG gme‐665‐ab) and Amazon Web Services (https://aws.amazon.com/). First, we removed sequencing adapters and low‐quality bases (phred score < 30) with Trimmomatic v0.38 (Bolger et al., 2014). We mapped the trimmed reads to a chromosome‐level caribou reference genome (Taylor et al., 2022) which has an N50 score of 64.42 Mb using Bowtie2 v2.3.0 (Langmead & Salzberg, 2012).

We used Samtools v1.5 (Li et al., 2009) to convert the SAM files to BAM files and to sort the BAM files. We then removed duplicate reads and added read group information to each BAM file with Picard v2.17.3 (Broad Institute, n.d.). We used Sambamba v0.8.0 (Streit et al., 2013) to retain only primary alignments and BamUtil v1.0.14 (https://github.com/statgen/bamUtil) to clip overlapping regions. We used Samtools to remove bases with a mapping quality (q) lower than 20 and index the BAM files. We checked the quality of each BAM file using FastQC v0.11.8 (Andrews, 2010). Finally, we used Samtools to produce alignment statistics (flagstat) and to calculate the depth of coverage across each genome.

We used the GATK v4.0.2 (McKenna et al., 2010) Haplotype Caller to produce Genomic variant call format (GVCF) files for each caribou. We then used CombineGVCFs and GenotypeGVCFs in GATK to combine and genotype the GVCFs, producing grouped VCF files. We used VCFtools v0.1.14 (Danecek et al., 2011) to select scaffolds and perform filtering. Although the reference genome used in this study does not have a sex chromosome characterized, several regions on Scaffold 36 had genes linked with sex chromosomes (Liu et al., 2019). Thus, we selected the 35 largest scaffolds (representing >99% of the genome) to focus our analyses on large autosomes and performed additional filtering: we removed sites with a depth <2 or >60, indels, non biallelic sites, low‐quality genotype calls (GQ < 20), and genotypes with more than 50% missing data (henceforth: filtered VCF). Finally, we produced a more strictly filtered version that contained no missing data (henceforth: strictly filtered VCF).

We attempted to retain as many informative sites as possible, as strict loci filtering can lead to irresolute conclusions and bioinformatics tools are becoming reliable when performing under randomly distributed missing data (Hodel et al., 2017; Huang & Lacey Knowles, 2016). Minor allele frequency (MAF) and Hardy–Weinberg equilibrium (HWE) estimates are conventionally calculated to identify putative sequencing artifacts (Chang, 2020); however, excluding loci based on these parameters can lead to allelic dropout. Both parameters are highly dependent on sampling size and the population of origin, and can represent true evolutionary signals (Pearman et al., 2022). Outlier and linkage disequilibrium (LD) scans search for loci with allelic frequencies out of neutral, and therefore random, expectations. Thinning for LD is likely to exclude many diagnostic markers, decreasing the power of the analyses, including ROH identification (Meyermans et al., 2020). Given divergent population histories and the small number of samples representing each population, we did not filter our data for MAF, HWE, or LD; however, we attempted to account for LD in our population history analyses as described below.

### 2.3 Genomic population structure

We explored population structure using the two filtered VCF files. We used Atlas (Link et al., 2017) to convert the filtered VCF file to a Beagle file for NGSadmix (Skotte et al., 2013). We then used NGSadmix v32 to explore population groupings among individuals (*K* = 2–9). We conducted 10 arrays at each *K* value and then used R to plot the outputs (Figure 2) and compare the log likelihood values across runs to select the best supported number of populations (*K*). Specifically, we used the Cluster Markov Packager Across *K* from Evanno (Evanno et al., 2005) via an R script provided by Bay et al. (2021) to select the best *K* value by dividing the mean log likelihood of each *K* by the standard deviation (Table S2).

**FIGURE 3:**
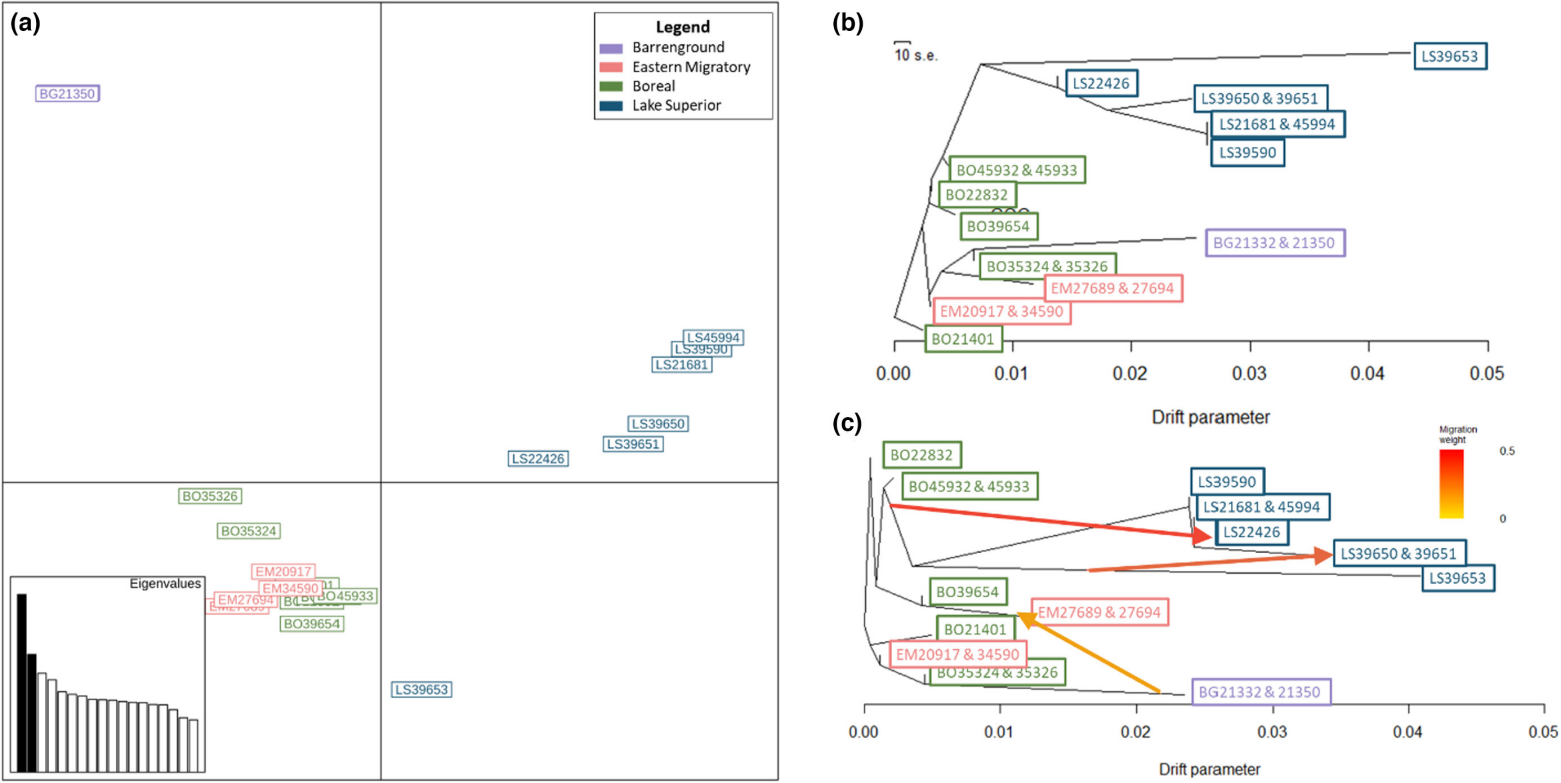
(a) Principal Component Analysis (PCA) visualizing genomic variation among caribou (*N* = 20). (b) Treemix plot without migration showing evolutionary relationships (*k* = 1000). Branch lengths indicate drift estimates. (c) Treemix migration model with the best support (*k* = 1000). Arrows indicate the direction of migration or gene flow; arrow colors indicate the strength of migration.

We conducted Principal Component Analyses (PCA; Figure 3a) in R v4.0.2 (R Core Team, 2020) using the strictly filtered VCF containing no missing data. We used Stacks v2.60 (Catchen, 2013) to convert the filtered VCF file to input for Treemix v1.1. To account for possible linkage, we performed analyses with different sized groupings of SNPs (*k* = 500, 1000, and 2000). We created evolutionary trees (Figure 3) with and without migration events (*m* = 0–7). We performed 10 arrays for each parameter and plotted the outputs in R. We then used the OptM package in R (Fitak, 2021) to select the migration model with the most support.

### 2.4 Genomic diversity and inbreeding

We calculated individual inbreeding coefficients (*F*) based on observed and expected heterozygosity using VCFtools (Danecek et al., 2011) with the strictly filtered VCF file (Table S3). We also quantified inbreeding as the amount of genome in ROH using PLINK v1.90b4.6 (Chang et al., 2015) and ROHan (Renaud et al., 2019). PLINK examines SNP data using a window‐based observational approach to identify ROH segments, which are homozygous genomic regions where an individual has received the same copy of an allele from both parents due to inbreeding (Meyermans et al., 2020). Conversely, ROHan combines a local Bayesian model and hidden Markov model (HMM) to identify ROH from individual mapped genomes (Renaud et al., 2019).

We assessed the robustness of our results by examining multiple parameters with two size categories and different rates of the number of heterozygous sites allowed under both ROH methods. For all PLINK analyses, we used the strictly filtered VCF file and did not filter for MAF nor LD following recommendations from Meyermans et al. (2020). We selected parameters based on similar investigations of non‐model chromosome‐level genome assemblies (e.g., Duntsch et al., 2021; Lavanchy & Goudet, 2023; Martin et al., 2023; von Seth et al., 2021). Specifically, we applied the following “strict” parameters: homozyg‐window‐snp 100, homozyg‐window‐het 1, homozyg‐het 5, homozyg‐gap 200, homozyg‐density 50, and homozyg‐snp 100. We also conducted PLINK analyses with more “relaxed” parameters: homozyg‐window‐snp 50, homozyg‐window‐het 2, homozyg‐het 10, homozyg‐gap 1000, homozyg‐density 100, and homozyg‐snp 50. Both sets of parameters included homozyg‐window‐threshold 0.05. Following the same approach as Martin et al. (2023), we applied these parameters under two ROH size categories by using homozyg‐kb to identify ROH > 250 kb and >1 Mb in length. *F*_ROH_ was then calculated for each individual as the total length of ROH divided by the length of the 35 chromosomes examined (Figure S5).

For ROHan analysis, we used the unfiltered BAM files as the program takes base quality into account (Renaud et al., 2019). Under the relatively “strict” parameters, we allowed 5 × 10^−5^ heterozygous sites within ROH (‐‐rohmu 5e‐5), and the more “relaxed” parameters allowed 5 × 10^−4^ heterozygous sites within ROH. For all ROHan analyses, we specified a transition/transversion ratio of 2.09 based on a calculation from the strictly filtered VCF file (‐‐tstv 2.09). Similar to our PLINK approach, we conducted analyses under both sets of parameters with 250 kb (‐‐size 250,000) and 1 Mb windows. The percent of genome in ROH reported by ROHan was converted to a proportion to represent *F*_ROH_ (Figure S6).

Finally, to measure genetic diversity, we calculated genome‐wide heterozygosity (Watterson's θ), producing two estimates for each individual: heterozygosity across all genomic regions and excluding regions in ROH in ROHan (Renaud et al., 2019). For this analysis we used 250 kb windows with a heterozygosity rate of 5 × 10^−4^ tolerated within ROH.

## 3 RESULTS

### 3.1 Genome quality control

All individual BAM files passed quality control with FastQC and Samtools flagstat. The final filtered BAM files had an average depth of 29× (Table 1). We created two versions of the grouped VCF to retain as many informative sites as possible: the filtered version contained 23,859,411 SNPs; and the strictly filtered VCF contained 9,338,805 SNPs.

### 3.2 Population structure

We first explored genomic structure among populations of caribou ecotypes. We investigated population groupings with NGSadmix and found the best supported model was *K* = 2 (Figure 2), which had the highest log likelihood value and 100% convergence across runs (Table S2). We found barren‐ground, eastern migratory, and boreal caribou from the continuous range were assigned to the first cluster, although the eastern migratory and boreal samples from Ontario also shared a small proportion of assignment to the second cluster (Figure 2). The Lake Superior caribou were mostly assigned to the second cluster, except for the individual from Pukaskwa National Park which was split between the two groups (53%). The next best supported model was *K* = 3 (Figure S1), which also indicated the Lake Superior caribou cluster together, with Pukaskwa National Park and one other sample showing mixed assignment. The eastern migratory Ontario and boreal continuous range caribou were assigned together. The third cluster was comprised of barren‐ground and eastern migratory samples from the George River herd, with a small proportion of mixed assignment observed in boreal Manitoba and a boreal sample from Cochrane, Ontario (Figure S1).

The Principal Component Analysis (PCA) revealed genomic groupings among samples (Figure 3a, Figure S3). We retained 19 Principal Components (PCs); PC1 and PC2 (Figure 3a) collectively explained 20% of the cumulative variance (Figure S2). We plotted comparisons of PCs 1–4 (Figure S3). Our results distinguished the barren‐ground caribou from the other ecotypes present in the study, whereas eastern migratory caribou and boreal caribou from the continuous range grouped together (Figure 3a, Figure S3). The Lake Superior caribou largely grouped together, with the exception of the sample from Pukaskwa National Park. The Treemix results were consistent throughout iterations, regardless of the SNP grouping size (*k*‐value), and revealed the Lake Superior caribou on a branch together, with Pukaskwa National Park representing an older branch based on its basal position, which has experienced considerable drift as indicated by the drift parameter (Figure 3). The best supported Treemix model across all k‐values indicated three migrations (Figure S4); notably, all migrations originated from basal placements in the tree (rather than branch tips), which indicates the migration occurred historically or from a closely related unsampled population (Decker et al., 2014). We detected migration from Pukaskwa National Park to Michipicoten Island, from the nearby boreal continuous range (Nipigon) to Pic Island, and from barren‐ground into eastern migratory caribou from the George River herd (Figure 3c).

### 3.3 Genomic diversity and inbreeding

We estimated inbreeding as the inbreeding coefficient (*F*) and the proportion of the genome in ROH (*F*_ROH_; Table S3). We found that the lowest inbreeding coefficients produced by VCFtools were observed in the lowest coverage genomes (10×), and these findings had the lowest concordance with the *F*_ROH_ estimates from other methods; however, at higher coverages (>15×, *N* = 16) the individual inbreeding estimates corroborated with other methods (Table S3).

We identified ROH using two methods, under two sets of parameters, and at different size scales to identify shorter ROH associated with historical inbreeding, and longer ROH indicating recent inbreeding. Not surprisingly, we found fewer, but longer ROH when analyses are restricted to a larger size class with both methods (Figures S5 and S6). For instance, when PLINK is restricted to ROH > 1 Mb, we detect zero ROH in several individuals, even under relaxed parameters (Figure S5). Under the strict ROHan parameters, we detected little to no ROH in any individual (Table S3, Figure S6), underscoring the importance of examining results under multiple methods and parameters.

**FIGURE 4:**
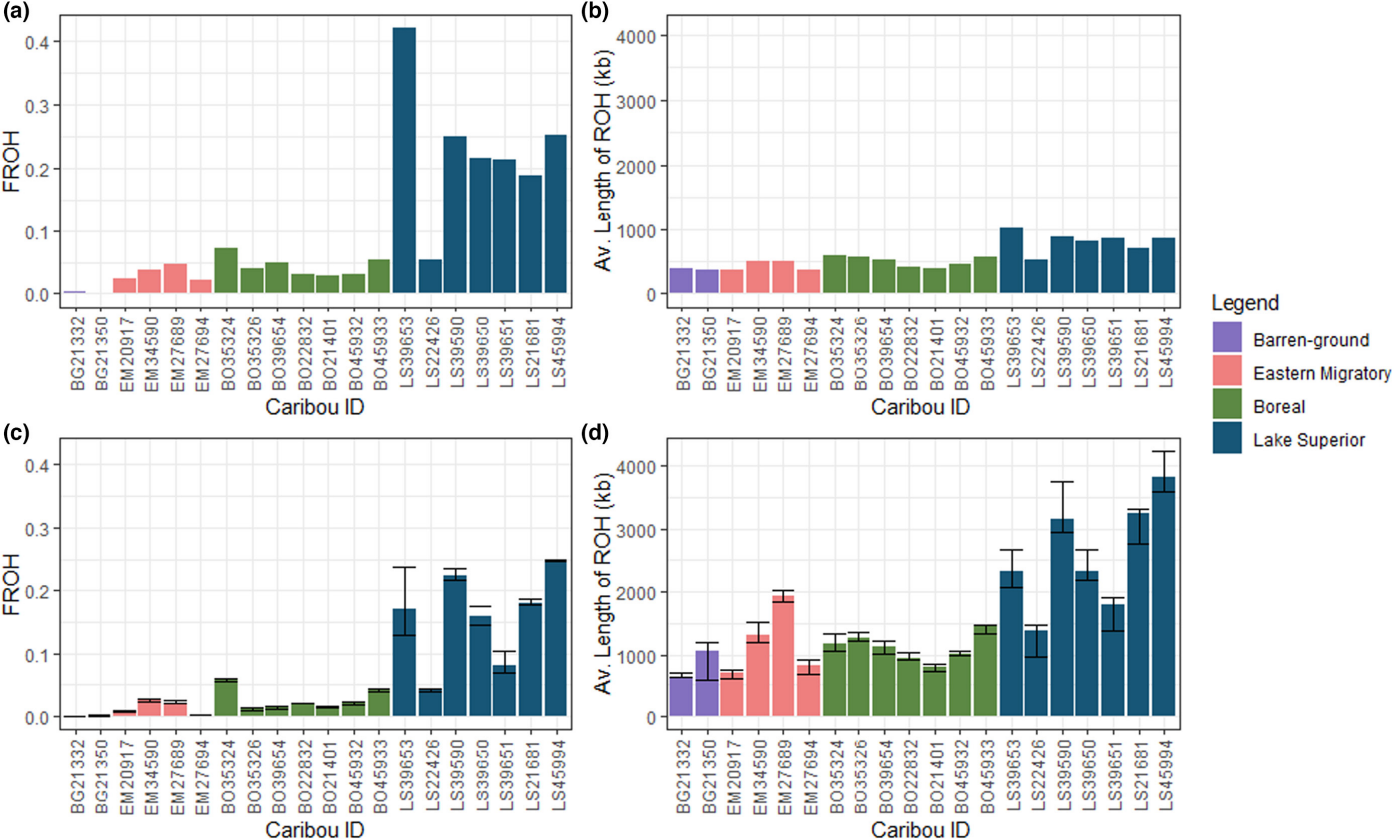
Inbreeding estimates in caribou based on Runs of Homozygosity (ROH) identified with PLINK (a, b) and ROHan (c, d) under the 250 kb size class. (a, c) *F*_ROH_ indicates the proportion of the genome classified as ROH. (b, d) Reflects the average length of ROH in kilobases, where shorter ROH indicate historical inbreeding and longer ROH indicate recent inbreeding. Error bars (c, d) represent the minimum and maximum estimates produced by the hidden Markov model in ROHan.

Across the methods and parameters we explored, barren‐ground caribou consistently had the lowest inbreeding levels, and the highest inbreeding estimates were observed in caribou from the Lake Superior range (Table S3, Figures S5 and S6). These caribou had the highest inbreeding coefficents (*F*), the largest proportion of the genome in ROH (*F*_ROH_), and the ROH were notably long, indicating recent inbreeding (Figure 4). We found an abundance of ROH in the Lake Superior caribou under both size classes (250 kb and 1 Mb; Figures S5 and S6), suggesting both recent and historical inbreeding has occurred. Notably, one individual from Pic Island had lower ROH estimates than the other Lake Superior caribou, reflecting values similar to the boreal caribou from the continuous range (Figures S5 and S6). This may be the result of low levels of gene flow with the continuous range (Figure 2), low levels of drift in comparison to other Lake Superior caribou (Figure 3), or historically lower inbreeding levels as the sample was collected prior to 2008 (Table 1). All methods corroborated that the inbreeding levels in eastern migratory and boreal caribou from the continous range are higher than those observed in barren‐ground and lower than Lake Superior with little variation among individuals.

**FIGURE 5:**
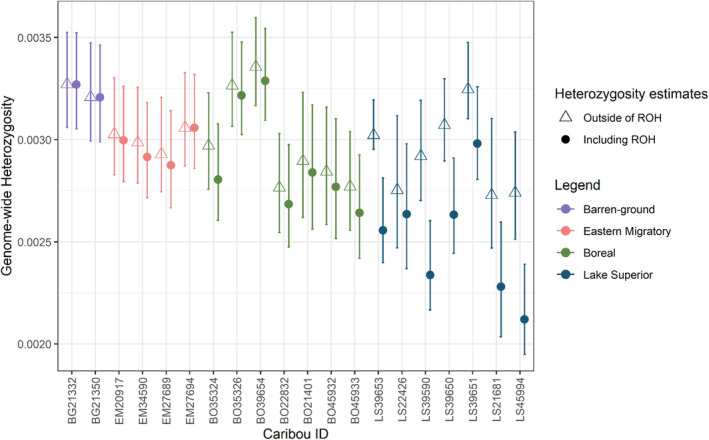
Individual genetic diversity in caribou, calculated as genome‐wide heterozygosity (Watterson's θ). Heterozygosity was calculated across the whole genome (including ROH; solid circles) and excluding regions in ROH (hollow triangles), using 250 kb windows and allowing a heterozygosity rate of 0.0005 within ROH. Error bars indicate the minimum and maximum estimates of the hidden Markov model.

The lowest genomic diversity estimates, calculated as genome‐wide heterozygosity, were observed in caribou from Lake Superior; however, some caribou from the Lake Superior range had relatively high heterozygosity estimates, with values similar to those observed in the continuous boreal range (Figure 5). Across all samples, most individuals showed no difference in heterozygosity inside and outside ROH, which is not surprising as many caribou had only a small amount of ROH identified. However, the Lake Superior caribou showed notably higher heterozygosity outside of ROH (Figure 5).

## 4 DISCUSSION

We sampled caribou from the trailing edge of Ontario's caribou range, and as predicted, these caribou exhibited high levels of inbreeding relative to caribou in the continuous range farther north (e.g., Hampe & Petit, 2005). The Lake Superior caribou also exhibited evidence of differentiation from caribou in the continuous range and low within‐population genomic diversity. The Lake Superior range contains Ontario's southernmost caribou populations, which have become small and isolated from other caribou in Ontario through anthropogenic range contraction (Schaefer, 2003; Vors et al., 2007). We found an abundance of short (>250 kb) and long (>1 Mb) ROH, indicating both historical and recent inbreeding has occurred (Figure S5). All of the other caribou populations investigated had comparatively low levels of inbreeding (Figure 4) regardless of evolutionary origins (NAL or BEL lineage; Klutsch et al., 2012; Polfus et al., 2017).

Broadly, the population groupings revealed by our analyses did not clearly reflect current management designations (DUs). Barren‐ground caribou are distinct from the other populations sampled in this study (Figure 3), which was predicted as they are the only samples from the BEL included. However, within the NAL, there is little distinction between eastern migratory and boreal caribou based on variation across the whole genome (Figure 3a). Our results indicate that caribou from the Lake Superior range group together but not closely with other boreal caribou from the continuous range (Figures 2 and 3a). We observed some evidence that this differentiation is due to the isolation of Lake Superior caribou from the continuous range, but further research should explore the importance of local adaptation in these island caribou.

### 4.1 Population structure

Our evolutionary tree revealed that caribou in the Lake Superior range form a consistent group that branches from the nearby continuous boreal range, with Pukaskwa National Park representing a basal branch (Figure 3). We detected evidence of gene flow from the continuous boreal range to the Lake Superior range, confirming a previous study that suggested remnant genetic connectivity between Lake Superior and the continuous range (Drake et al., 2018). We also detected weak migration or gene flow from barren‐ground caribou into eastern migratory caribou from George River, which is not surprising as previous research has indicated that the eastern migratory ecotype was formed by historical introgression between barren‐ground and boreal caribou (Klütsch et al., 2016).

The PCA and Treemix analyses revealed barren‐ground caribou are distinct from the other populations sampled in this study (Figure 3); however, we found little distinction between eastern migratory and boreal caribou. These results are consistent with other genomic research that revealed eastern migratory and boreal caribou from NAL cannot be divided into monophyletic lineages (Taylor et al., 2022). Further, when the samples are assigned to two population clusters (Figure 2), barren‐ground, eastern migratory, and boreal caribou from the continuous range group together, which is likely due to historical introgression among these ecotypes (Taylor et al., 2020).

Our results revealed low differentiation within the Lake Superior range but high levels of differentiation from the continuous range, which is predicted for rear edge populations (Hampe & Petit, 2005). In fact, we observed a greater distinction between the Lake Superior range and continuous range boreal caribou than we did between the boreal and eastern migratory ecotypes. We did not find evidence of high regional diversity among caribou within the Lake Superior range, which can occur in isolated trailing edge populations due to high levels of genetic drift and a lack of gene flow among patches (Hampe & Petit, 2005). Increased sampling of other patches along the trailing edge is likely required to investigate divergent drift patterns. However, our results suggest some connectivity exists, or recently existed, among Lake Superior islands and coastal regions. Previous research has also suggested connectivity exists within the Lake Superior range, as a caribou radio‐collared on the Slate Islands traveled to Pukaskwa National Park, following the nearshore past other sites included within our study, near Neys Provincal Park and Pic Island (Bergerud, 1985; Bergerud et al., 2007). The previous studies suggested that in the past these caribou have made long movements but always stayed near the Lake Superior shore (Bergerud, 1985; Bergerud et al., 2007), which is supported by our findings demonstrating low differentiation within the Lake Superior range with high differentation from the continuous range.

The boreal and eastern migratory ecotypes both originate from the NAL and share extensive areas of habitat overlap, particularly in winter (COSEWIC, 2017b). However, these ecotypes are managed as distinct DUs based on differences in behavior and life history strategies: eastern migratory caribou aggregate on the tundra during calving and are the only group of NAL caribou to migrate (COSEWIC, 2017b). Conversely, boreal caribou remain within the forest year‐round and avoid conspecifics during calving, instead relying on dense woods to avoid predation (COSEWIC, 2014a).

Interestingly, the long‐term persistence of caribou in the Lake Superior range is partially attributed to their calving strategy: instead of using the typical strategy of boreal caribou, who avoid wolf predation by using dense woodlots to space out from conspecifics when calving, the Lake Superior caribou use the shoreline and nearby islands to escape predation (Bergerud, 1985; Bergerud et al., 2014). Another factor encouraging caribou persistence in this range is the presence of protected areas (e.g., Pukaskwa National Park, Neys Provincial Park) with low levels of anthropogenic disturbance (Schaefer, 2003). The islands and protected coastal areas may provide refugia from the negative impacts of human encroachment. As negative human impacts spread, areas along the range periphery and remote islands are less impacted by anthropogenic disturbances and thus, represent patches where persistence is more likely than it is in the core range, providing valuable opportunities for conservation (Channell & Lomolino, 2000). Notably, the features demonstrated by the Lake Superior caribou, such as small population sizes, isolation from the core range, and associations with distinct habitat features can encourage local adaptation (Hampe & Petit, 2005). In fact, evolutionary theory suggests that peripheral populations face more diverse environmental conditions than central populations; and thus, are more likely to be preadapted to anthropogenic disturbances that pose a threat to the species across its entire range (Lomolino & Channell, 1998). Conversely, adaptive processes may be hindered by the high levels of inbreeding and drift experienced by the remaining caribou in the Lake Superior range.

### 4.2 Inbreeding histories

We found inbreeding estimates produced across methods varied in magnitude but generally corroborated on inbreeding ranks among individuals. Our results indicated inbreeding coefficient estimates produced by VCFtools may be unreliable for lower depths of coverage (Table S3); however, our data met the minimum requirements of 10× depth for PLINK and 5× depth for ROHan to produce reliable ROH estimates (Renaud et al., 2019). As the field of conservation genomics rapidly expands, we urge researchers to ensure their data meet the minimum requirements for inbreeding analyses, as a high density of SNPs is required for accurate ROH identification and reduced genome coverages result in an underestimation of *F*_ROH_ (Lavanchy & Goudet, 2023; Meyermans et al., 2020). Additionally, after assessing that the data meet the minimum depth and SNP density requirements, we suggest conducting analyses with multiple methods under different parameters to ensure the results are robust. In general, we observed a greater abundance but shorter ROH with PLINK than we did with ROHan (Figure 4). The reporting of shorter ROH could be due to differences in the input data or the underlying models. For instance, PLINK used sliding window observations, whereas ROHan used a HMM approach; PLINK examined high‐quality variant sites across the genomes, whereas ROHan examined all mapped sites, resulting in more continuous data (Renaud et al., 2019). Additionally, the specific PLINK parameters used, such as the SNP density requirements, may bias the results towards shorter ROH.

Caribou from the Lake Superior range consistently had higher inbreeding estimates than the other populations sampled (Figures S5 and S6). These small coastal and island populations are relatively isolated and have experienced several bottlenecks (Bergerud et al., 2007; Fletcher, 2017). The sample from Pukaskwa National Park demonstrated high levels of inbreeding, including notably long ROH indicating recent inbreeding. The caribou population in Pukaskwa National Park persisted at low levels for years and currently no caribou remain in the park; however, one of the last caribou recorded with wildlife cameras in the park had malformed antlers, which was suggested to be evidence of inbreeding depression (Drake et al., 2018). The high *F*_ROH_ values observed in the Lake Superior caribou reflect their overall inbreeding levels, whereas the combination of short and long ROH likely reflects the historical and recent bottlenecks experienced by these populations.

Despite consistently elevated inbreeding levels, we found several caribou from the Lake Superior range maintained relatively high levels of genetic diversity. The lowest diversity levels correspond to caribou from the Slate Islands with high ROH estimates; however, our results indicated relatively high diversity has been maintained outside of ROH (Figure 5). This suggests genetic diversity may be maintained by natural selection in genomic regions where variation is important (Hedrick & Garcia‐Dorado, 2016; Selli et al., 2021).

Given the consistently high inbreeding levels observed in the Lake Superior range compared to the other populations, it is possible that divergent inbreeding histories are further driving the observed genomic differences between populations. However, one of the individuals with the highest inbreeding levels and the highest drift estimate, from Pukaskwa National Park, showed more similarities to the continuous range than the other Lake Superior caribou did, although these results may also suggest the sample from Pukaskwa National Park was more similar to the continuous range than it was to the other Lake Superior samples (Figures 2 and 3). If the distinctions between populations were largely driven by inbreeding or drift, we would expect the individual with the highest inbreeding and drift estimates to show the greatest distinction, whereas the Lake Superior samples with comparatively lower inbreeding and drift levels should demonstrate more similarities with the continuous range. Notably, the population structure patterns observed (Figure 2) may be indicative of three different evolutionary scenarios, which can be difficult to disentangle (Garcia‐Erill & Albrechtsen, 2020; Lawson et al., 2018). Specifically, the patterns demonstrated by the Lake Superior caribou may be due to multiple recent bottlenecks, such as a bottleneck when the Pukaskwa National Park population diverged followed by a subsequent bottleneck when the other Lake Superior caribou diverged. Alternatively, the population assignment observed in the sample from Pukaskwa National Park may be reflecting recent admixture or ghost admixture from a historical lineage that has been lost or was not sampled (Garcia‐Erill & Albrechtsen, 2020; Lawson et al., 2018). Our results may be affected by uneven sampling (Puechmaille, 2016), especially as we are using a single sample from some locations. Future research should strive to sequence additional genomes to allow for more even sampling design; although, this may be challenging for some regions where caribou are now locally extinct (e.g., Pukaskwa National Park).

Significant efforts have been invested in the continued persistence of the Lake Superior caribou populations, including multiple relocations between islands (Bergerud et al., 2007; Ontario Ministry of Natural Resources and Forestry, 2018). Given the small number of caribou remaining and high degree of inbreeding, we recommend that future management decisions take inbreeding into consideration. Understanding individual inbreeding levels may be especially important in the context of relocations, and should be considered and monitored when reestablishing or supplementing populations (Scott et al., 2020). Thus, we are further investigating the level of inbreeding using a larger sample size from the different populations with a focus on caribou that have been recently relocated following rapid declines.

### 4.3 Conclusions

We used high‐coverage whole genomes to delineate population structure and inbreeding histories in caribou from populations representing divergent evolutionary histories, differing in population size and extent of isolation. We found eastern migratory caribou and boreal caribou from the continuous range broadly cluster together under population genomic models. We found caribou from the Lake Superior range form a distinct group; however, we also detected evidence of gene flow between Lake Superior and the continuous range of boreal caribou. Specifically, we identified a nearby population in the continuous range with evidence of shared ancestry and historical gene flow to the Lake Superior range, which could be used to inform future management if restoring connectivity between the two ranges is a priority (Armstrong et al., 2010), and deemed appropriate given the potential for local adaptation.

We found the lowest levels of inbreeding in barren‐ground caribou and relatively low inbreeding estimates in eastern migratory and boreal caribou from the continuous range. We observed consistently elevated inbreeding estimates in the Lake Superior populations, which have experienced historical bottlenecks, recent declines, and become increasingly isolated due to recent range contraction (Bergerud et al., 2007, 2014; Schaefer, 2003). We observed an abundance of both long and short ROH in these isolated populations, confirming both historical and recent inbreeding has occurred. Given the results of our study, the high levels of inbreeding in the Lake Superior caribou may be further driving the observed distinctions between populations. To determine the significance of the observed population structure, future research should attempt to investigate local adaptation.

## AUTHOR CONTRIBUTIONS

**Kirsten Solmundson:** Conceptualization (lead); data curation (lead); formal analysis (lead); funding acquisition (lead); investigation (lead); methodology (lead); project administration (lead); visualization (lead); writing – original draft (lead); writing – review and editing (lead). **Jeff Bowman:** Conceptualization (supporting); data curation (supporting); funding acquisition (supporting); project administration (supporting); resources (supporting); supervision (lead); writing – review and editing (supporting). **Micheline Manseau:** Conceptualization (supporting); data curation (supporting); funding acquisition (supporting); project administration (supporting); resources (supporting); supervision (supporting); writing – review and editing (supporting). **Rebecca S. Taylor:** Formal analysis (supporting); investigation (supporting); methodology (supporting); writing – review and editing (supporting). **Sonesinh Keobouasone:** Data curation (supporting); formal analysis (supporting); resources (supporting); software (supporting). **Paul J. Wilson:** Conceptualization (supporting); data curation (supporting); funding acquisition (supporting); project administration (supporting); resources (supporting); supervision (lead); writing – review and editing (supporting).

## Supporting information


Data S1:

## Data Availability

The raw reads are available at the National Centre for Biotechnology Information (NCBI) under the BioProject accession numbers PRJNA 634908 and PRJNA 984705. Spatial layers for mapping the ranges of Designatable Units were provided by the Committee on the Status of Endangered Wildlife in Canada. Annotated code for all analyses can be found at: https://github.com/ksolmundson/PopGenomics.
