# Supplementary material for: Genomic population structure and inbreeding history of Lake Superior caribou
Source: Ecol Evol. 2023 Jul 7;13(7):e10278. doi: 10.1002/ece3.10278 (PMC10326607; doi:10.1002/ece3.10278)
Supplement: Supplementary file 1 — Data S1: [file ECE3-13-e10278-s001.docx]

**Supplemental Information for:**

**Whole genomes reveal caribou population structure and inbreeding histories**

Kirsten Solmundson, Jeff Bowman, Micheline Manseau, Rebecca S. Taylor, Sonesinh Keobouasone, Paul J. Wilson

**Table of Contents:**

| **Table S1** | Page 2 |
| --- | --- |
| **Table S2** | Page 3 |
| **Table S3** | Page 3 |
| **Figure S1** | Page 4 |
| **Figure S2** | Page 4 |
| **Figure S3** | Page 5 |
| **Figure S4** | Page 6 |
| **Figure S5** | Page 7 |
| **Figure S6** | Page 8 |

**Table S1.** Sample ID reference numbers and genomic reference numbers to access raw sequence data used in this study.

| Sample ID | Subspecies | Sample type | Genome accession number |  |
| --- | --- | --- | --- | --- |
| BG21332 | *R. t. groenlandicus* | Previously sequenced | PRJNA634908 |  |
| BG21350 | *R. t. groenlandicus* | Previously sequenced | PRJNA634908 |  |
| EM20917 | *R. t. caribou* | Previously sequenced | PRJNA634908 |  |
| EM34590 | *R. t. caribou* | Previously sequenced | PRJNA634908 |  |
| EM27689 | *R. t. caribou* | Previously sequenced | PRJNA634908 |  |
| EM27694 | *R. t. caribou* | Previously sequenced | PRJNA634908 |  |
| BO35324 | *R. t. caribou* | Previously sequenced | PRJNA634908 |  |
| BO35326 | *R. t. caribou* | Previously sequenced | PRJNA634908 |  |
| BO39654 | *R. t. caribou* | Previously sequenced | PRJNA634908 |  |
| BO22832 | *R. t. caribou* | Hair | PRJNA984705 |  |
| BO21401 | *R. t. caribou* | Fecal | PRJNA984705 |  |
| BO45932 | *R. t. caribou* | Muscle | PRJNA984705 |  |
| BO45933 | *R. t. caribou* | Muscle | PRJNA984705 |  |
| LS39653 | *R. t. caribou* | Hide | PRJNA984705 |  |
| LS22426 | *R. t. caribou* | Fecal | PRJNA984705 |  |
| LS39590 | *R. t. caribou* | Previously sequenced | PRJNA634908 |  |
| LS39650 | *R. t. caribou* | Muscle | PRJNA984705 |  |
| LS39651 | *R. t. caribou* | Muscle | PRJNA984705 |  |
| LS21681 | *R. t. caribou* | Fecal | PRJNA984705 |  |
| LS45994 | *R. t. caribou* | Antler | PRJNA984705 |  |

**Table S2.** Model probabilities based on the mean and SD of log likelihood values produced by NGSAdmix across an array of 10 runs. The model with the highest probability (K=2) is the best supported value of K.

| K=2 | K=3 | K=4 | K=5 | K=6 | K=7 | K=8 | K=9 |
| --- | --- | --- | --- | --- | --- | --- | --- |
| Inf | 1385.0690 | 248.9362 | 308.2456 | 343.2897 | 140.3178 | 133.4855 | 111.7235 |

**Table S3.** Inbreeding estimates calculated using VCFtools, PLINK, and ROHan with a size class of 250 kb. We used the strictly filtered VCF file to calculate the inbreeding coefficient (F) in VCFtools and to identify ROH using PLINK with two sets of parameters (as specified in Methods). We also identified ROH in the individual BAM files using ROHan with two sets of parameters.

| Sample ID | Mean Depth | F _(VCFtools)_ | F_ROH (PLINK strict)_ | F_ROH (PLINK relaxed)_ | F_ROH_ _(ROHan strict)_ | F_ROH_ _(ROHan relaxed)_ |
| --- | --- | --- | --- | --- | --- | --- |
| BG21332 | 38x | -0.03 | 0.002 | 0.003 | 0 | 0.001 |
| BG21350 | 38x | -0.03 | 0.001 | 0.002 | 0 | 0.002 |
| EM20917 | 36x | 0 | 0.015 | 0.023 | 0 | 0.009 |
| EM34590 | 37x | 0.02 | 0.029 | 0.037 | 0 | 0.025 |
| EM27689 | 38x | 0.07 | 0.037 | 0.048 | 0 | 0.021 |
| EM27694 | 39x | 0.04 | 0.013 | 0.022 | 0 | 0.002 |
| BO35324 | 35x | 0.05 | 0.06 | 0.071 | 0 | 0.058 |
| BO35326 | 38x | -0.04 | 0.034 | 0.041 | 0 | 0.013 |
| BO39654 | 38x | 0.05 | 0.039 | 0.048 | 0 | 0.014 |
| BO22832 | 19x | 0.01 | 0.022 | 0.03 | 0.002 | 0.021 |
| BO21401 | 10x | -0.08 | 0.016 | 0.028 | 0.001 | 0.017 |
| BO45932 | 13x | -0.03 | 0.022 | 0.03 | 0.001 | 0.022 |
| BO45933 | 18x | 0.04 | 0.043 | 0.053 | 0.009 | 0.043 |
| LS39653 | 40x | 0.41 | 0.399 | 0.42 | 0 | 0.171 |
| LS22426 | 10x | -0.06 | 0.04 | 0.052 | 0.008 | 0.04 |
| LS39590 | 35x | 0.23 | 0.235 | 0.25 | 0 | 0.225 |
| LS39650 | 37x | 0.20 | 0.202 | 0.215 | 0 | 0.161 |
| LS39651 | 40x | 0.20 | 0.199 | 0.212 | 0 | 0.081 |
| LS21681 | 10x | 0.08 | 0.165 | 0.188 | 0.074 | 0.179 |
| LS45994 | 17x | 0.22 | 0.233 | 0.251 | 0.041 | 0.248 |


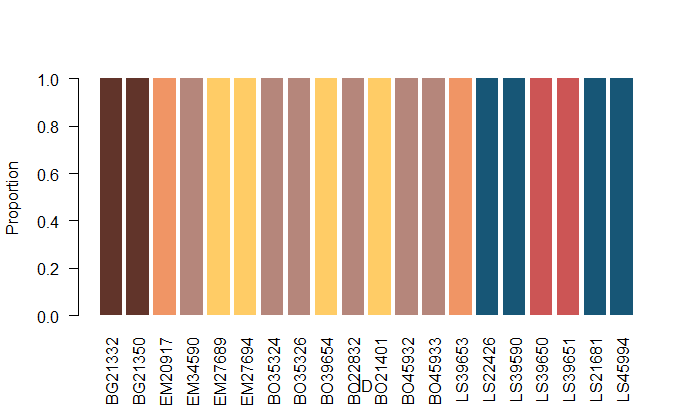

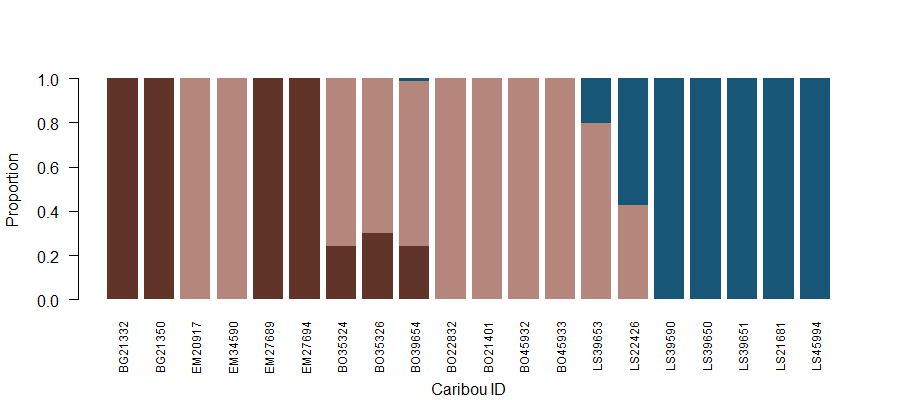


**Figure S1.** NGSAdmix plot for the next best supported K-values (3 and 6). Each bar represents an individual and colours represent population assignments.


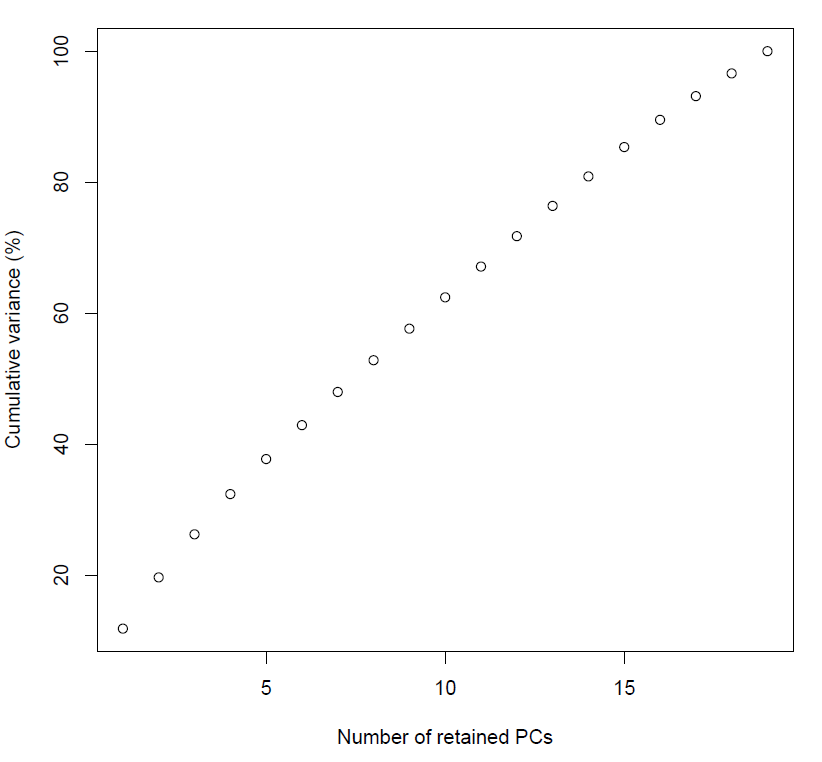


**Figure S2**. Cumulative variance for the 19 Principal Components of the PCA.


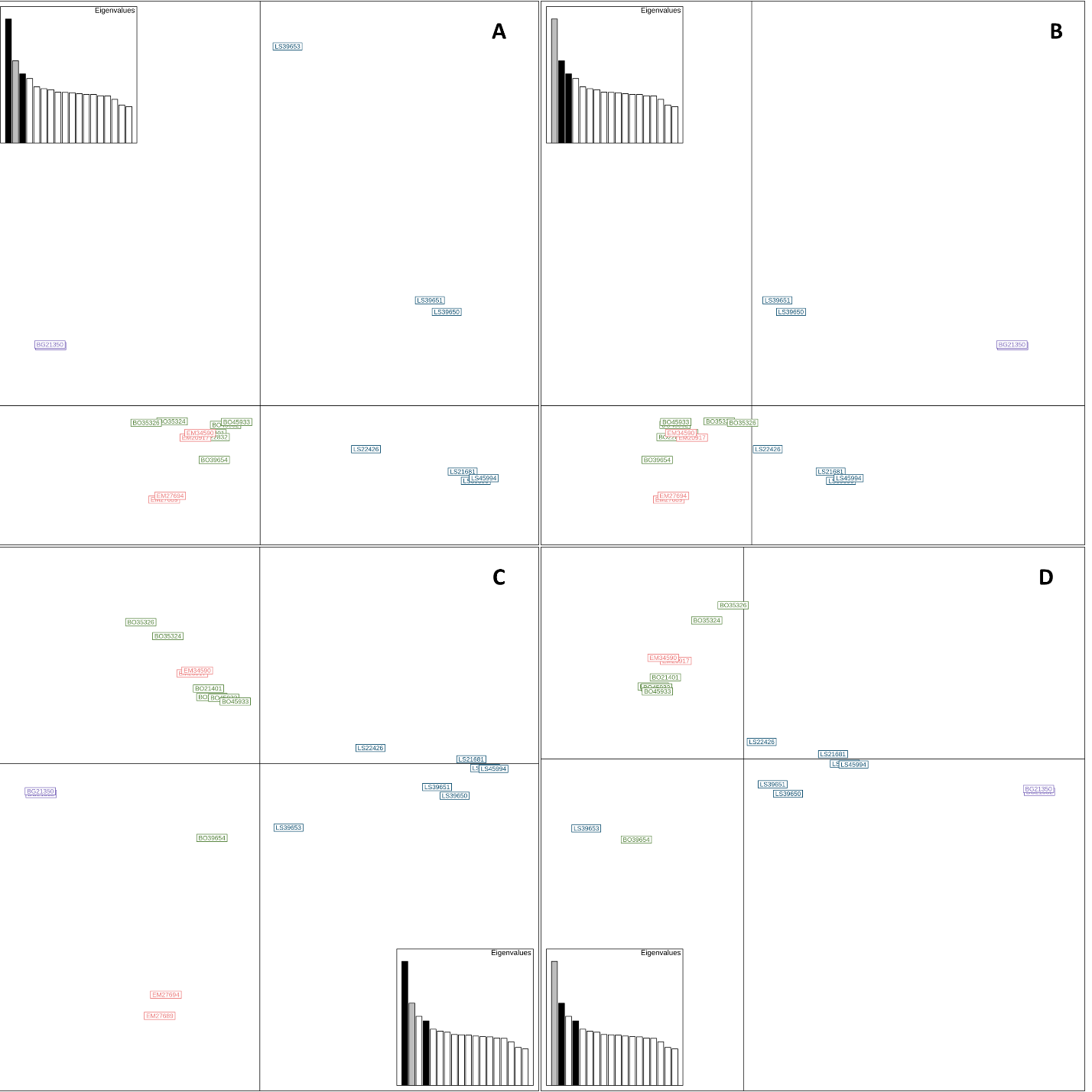


**Figure S3.** Additional Principal Component Axes: A) 1vs3 B) 2vs3 C) 1vs4 D) 2vs4


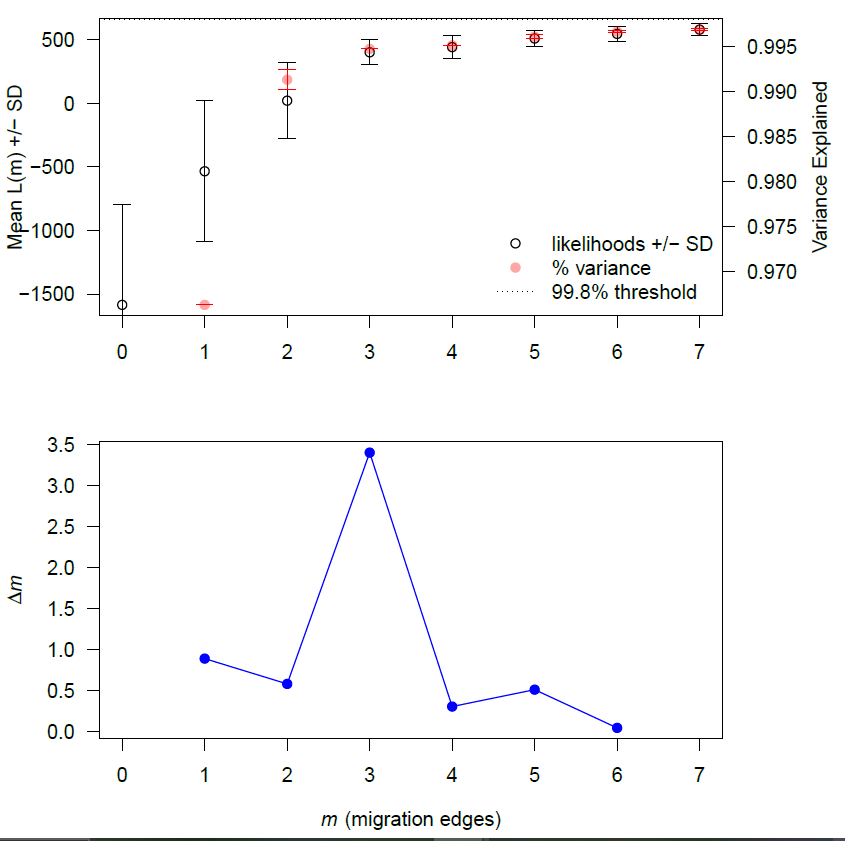


B

A

**Figure S4.** Comparison of Treemix migration models. We performed 10 iterations at k-values of 500, 1000, and 2000 for each migration model (m=0-7). The likelihood and SD values (A) and comparison of ∆m values (B) indicate the model with 3 migrations has the best support.


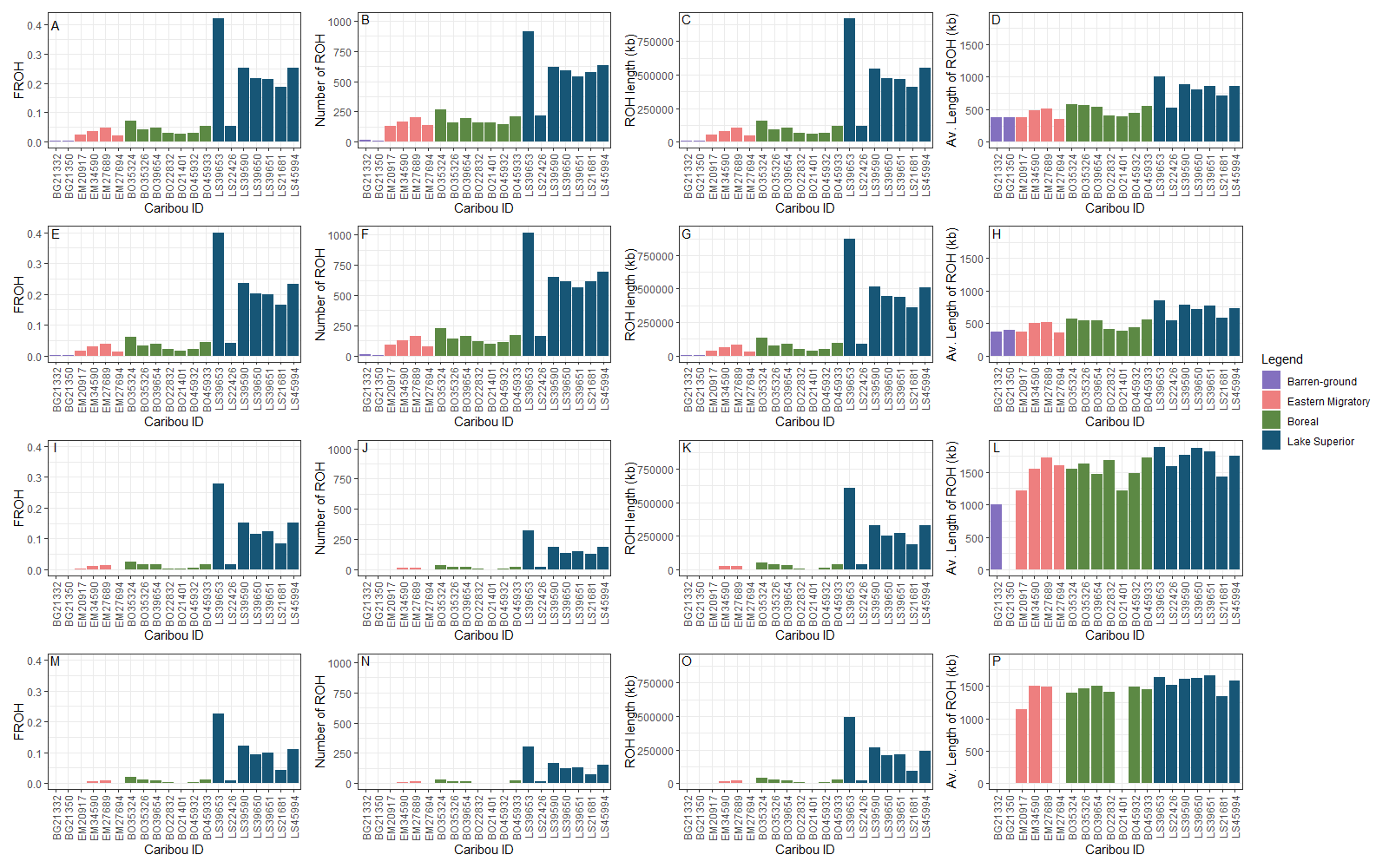


**Figure S5.** Runs of homozygosity identified with PLINK under different sets of parameters. A-D) relaxed parameters, >250kb ROH. E-H) strict parameters, >250kb ROH. I-L) relaxed parameters, >1Mb ROH. M-P) strict parameters, >1Mb ROH.


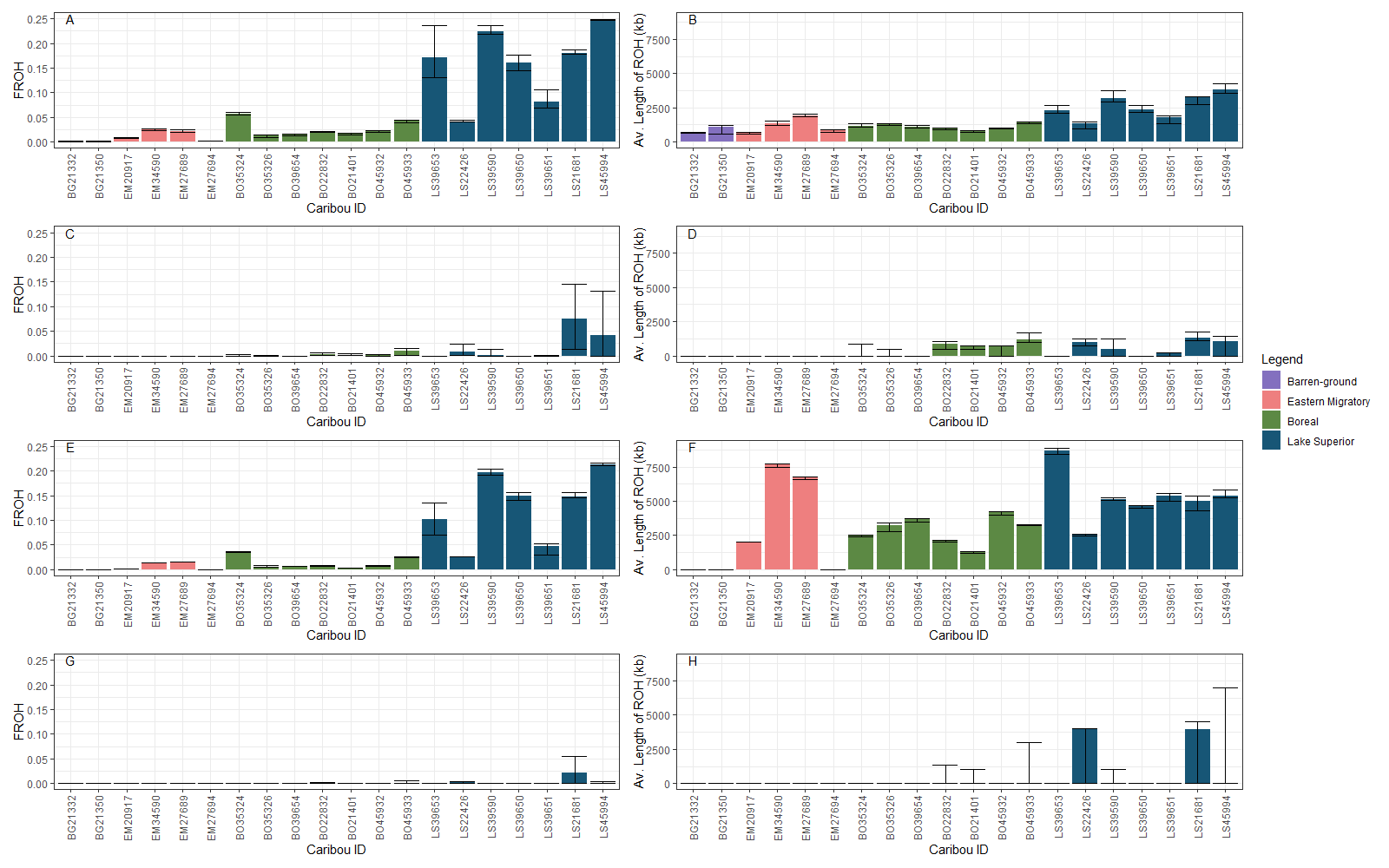


**Figure S6.** Runs of homozygosity identified with ROHan under different sets of parameters. We used window sizes of 250kb (A-D) and 1Mb (E-H) under relaxed (A,B,E,F) and more strict (C,D,G,H) parameters. Error bars indicate minimum and maximum estimates produced by the Hidden Markov Model.
